# Dynamics and chronology of *Mycoplasma hyopneumoniae* strain 232 infection in experimentally inoculated swine

**DOI:** 10.1186/s40813-021-00221-2

**Published:** 2021-06-30

**Authors:** Henrique M. S. Almeida, Marina L. Mechler-Dreibi, Karina Sonálio, Marcela M. Ferreira, Paulo E. B. Martinelli, Igor R. H. Gatto, Dominiek Maes, Hélio J. Montassier, Luís G. Oliveira

**Affiliations:** 1 São Paulo State University (Unesp), School of Agricultural and Veterinarian Sciences, Via de Acesso Prof. Paulo Donato Castellane s/n, Jaboticabal, SP 14884-900 Brazil; 2 Faculty of Veterinary Medicine, Ghent University, Ghent, Belgium

**Keywords:** Enzootic pneumonia, qPCR, Infectious diseases, Respiratory diseases, Diagnostics

## Abstract

Direct detection of *Mycoplasma hyopneumoniae* through molecular tools is a growing trend for early diagnosis, highlighting the importance of knowing *M. hyopneumoniae* dynamics in the respiratory tract upon infection. This study focused on monitoring the infection level and its effects in different anatomic sites of the respiratory tract of experimentally infected swine in four time-points post-infection. To this end, 24 pigs were allocated to either non-inoculated group (*n* = 8) or inoculated group (*n* = 16). On day 0 post-infection (dpi), animals of the inoculated group were intratracheally inoculated with *M. hyopneumoniae*. Nasal swabs were collected weekly for qPCR detection of bacterial shedding. At 14, 28, 42, and 56 dpi, four animals from the inoculated group and two from the control group were necropsied. Bronchoalveolar lavage fluid (BALF) and samples from three different anatomical tracheal sections (cranial - CT, medium - MT, lower - LT) were collected for qPCR and histopathology. Bacterial loads (qPCR) in tracheal samples were: 4.47 × 10^2^ copies∕μL (CT), 1.5 × 10^4^- copies∕ μL (MT) and 1.4 × 10^4^ copies∕μL (LT samples). *M. hyopneumoniae* quantification in BALF showed the highest load at 28 dpi (2.0 × 10^6^ copies∕ μL). Microscopic lesions in LT samples presented the highest scores at 56 dpi and were significantly correlated with the pathogen load on 14 dpi (0.93) and 28 dpi (0.75). The greatest bacterial load of *M. hyopneumoniae* in CT samples and BALF was registered at 28 dpi, and it remained high in BALF and LT throughout the 56 dpi. The pathogen was able to persist during the whole experimental period, however higher estimated quantification values were registered in the lower parts of the respiratory tract, especially at 56 dpi. These findings are important for improving diagnostics, treatment, and control measures of *M. hyopneumoniae* infection in swine herds.

## Background

*Mycoplasma hyopneumoniae* is the primary causative agent of enzootic pneumonia (EP) [1], a chronic disease characterized by dry, non-productive cough, high morbidity, low mortality, and losses in daily weight gain [2, 3]. The bacterium enters the host’s organism through the upper respiratory tract and adheres to the ciliated epithelium of the trachea, bronchus, and bronchi [4], disrupting the mucociliary defensive system and increasing infection susceptibility for secondary respiratory pathogens, which commonly results in co-infections, like the porcine respiratory disease complex (PRDC) [5].

*M. hyopneumoniae* is known for having a tropism for lower parts of the swine respiratory tract [6–9]. The population of this bacteria was shown to be 100 times higher in these sites when compared to the upper respiratory tract [6]. By assessing the lungs of experimentally infected pigs, *M. hyopneumoniae* was detected in bronchus and bronchioles as early as 7 days post-infection (dpi). Simultaneously, its direct detection in the alveolar macrophage and interstitial macrophages was only seen in the later stages of infection [7, 9].

Still, *M. hyopneumoniae* is notorious for its specific membrane proteins that adhere to the tracheal epithelial cilia during infection [10], inducing tissue inflammation and cell damage in vitro [11]. Thus, collection of tracheal secretions, using tracheobronchial swabs (deep tracheal catheters) and laryngeal swabs, have been proven to be sensitive and reliable diagnostic samples to detect *M. hyopneumoniae* infection in live animals [12, 13]. Consequently, it is very likely that the trachea is also an infection and multiplication site for *M. hyopneumoniae* [14]. However, as far as we know, there are no studies that looked into the distribution of the pathogen in the trachea throughout infection.

The knowledge about the dynamics and chronology of *M. hyopneumoniae* infection is essential for decision-making in the sampling methodology [15]. However, currently, there is little information regarding the bacterial load in different parts of the respiratory tract during infection. Considering the aforementioned fact, this study focused on reporting the infection dynamics upon experimental *M. hyopneumoniae* challenge and the associated effects in different respiratory tract sections of swine during a period of 56 dpi.

## Results

### Presence of clinical signs in inoculated piglets

The laryngeal swab and serum samples collected before inoculation were negative for *M. hyopneumoniae* DNA (qPCR) and specific *M. hyopneumoniae* antibodies (ELISA), respectively. While in the mock-inoculated animals, clinical signs compatible with EP were not detected, all inoculated animals presented clinical signs consistent with EP. The dry, non-productive cough was first noted at 10 dpi and thereafter observed in all inoculated animals. At the end of the experiment (56 dpi), at least one animal was still coughing. Beyond that, the animals did not show any clinical signs associated with other diseases.

### Detection of *M. hyopneumoniae* DNA in biological samples

#### qPCR parameters

All qPCR parameters followed the previously published recommendations described in the Minimum Information for Quantitative Real-Time qPCR Experiments (MIQE) [16]. The efficiency ranged from 92 to 98.3%, *R*^2^ ranged from 0.996 to 1.0, y-intercept ranged from 39 and 40.8, and the slope varied from − 3.36 to − 3.5.

#### Nasal swabs samples

Mock-inoculated animals remained negative during the whole study. The presence of *M. hyopneumoniae* DNA in nasal swab samples was detected from seven dpi (6∕16) onwards, and all animals, except for pig 2.4, presented at least one positive result across the study. Most animals showed positive results at 14 dpi and 21 dpi, 75% (12∕16) and 91.6% (11∕12), respectively. Bacterial shedding was shown to have an intermittent pattern, as reported in Table 1. The average Cq value obtained for each sampling day and the respective standard deviation are also shown in Table 1.

**Table 1:** Cq values obtained in qPCR of nasal swabs samples for detection of *M. hyopneumoniae* strain 232 in experimentally infected pigs throughout 56 dpi

Animal ID	**DPI**									
	**−7**	**0**	**7**	**14**	**21**	**28**	**35**	**42**	**49**	**56**
1.1	–	–	–	35.9						
1.2	–	–	36.3	37.5						
1.3	–	–	37.5	24.0						
1.4	–	–	–	21.4						
2.1	–	–	–	37.3	36.3	–				
2.2	–	–	–	35.5	35.0	–				
2.3	–	–	–	32.8	35.6	–				
2.4	–	–	–	–	–	–				
3.1	–	–	38.0	36.4	36.1	36.3	38.6	38.0		
3.2	–	–	–	–	36.6	–	–	38.1		
3.3	–	–	37.5	37.1	36.4	–	37.8	–		
3.4	–	–	37.5	–	34.9	35.9	39.4	36.3		
4.1	–	–	–	–	36.7	37.0	–	34.8	36.3	–
4.2	–	–	–	38.0	36.5	–	37.1	39.4	36.2	37.8
4.3	–	–	37.3	36.1	37.2	37.2	37.0	–	–	37.0
4.4	–	–	–	37.2	36.1	37.5	37.3	38.4	38.8	–
Cq Mean (±SD)			37.4 (±0.6)	31.1 (±5.5)	36.1 (±0.7)	36.8 (±0.7)	37.86(±1.0)	37.5(±1.7)	37.1(±1.5)	37.4(±0.6)
Total^a^	0∕16	0∕16	6∕16	12∕16	11∕12	5∕12	6∕8	6∕8	3∕4	2∕4

^a^Total expressed in positive∕total sampled animals

#### Tracheal samples

No positive results were obtained from the samples of mock-infected animals. The median of *M. hyopneumoniae* DNA quantification value in CT, MT, and LT samples at 14, 28, 42, and 56 dpi are shown in Table 2. Despite the numeric difference, no significant differences were found between *M. hyopneumoniae* burden in CT, MT and LT and dpi.

**Table 2:** Median and the range of quantification of P102 fragment copies∕μL of *M. hyopneumoniae* strain 232 in BALF, cranial, medium and lower trachea samples at four different dpi. Different superscript letters in the columns indicate significant differences at *p* < 0.05 between dpi in Dunn’s test

**Dpi**	**Sample**			
	**CT**	**MT**	**LT**	**BALF**
**14**	1.0 × 10^2^ (4.4 × 10^0^–1.23 × 10^4^)^a^	1.3 × 10^4^ (1.0 × 10^1^–1.9 × 10^4^)^a^	3.8 × 10^4^ (4.2 × 10^1^–7.58 × 10^4^)^a^	5.7 × 10^4^ (4.0 × 10^3^–1.2 × 10^5^)^b^
**28**	4.6 × 10^3^ (3.98 × 10^2^ - 1.53 × 10^6^)^a^	3.2 × 10^2^ (1.22 × 10^1^–1.05 × 10^3^)^a^	1.0 × 10^4^ (1.9 × 10^1^–1.15 × 10^5^)^a^	1.2 × 10^6^ (7.28 × 10^5^ -2.0 × 10^6^)^a^
**42**	1.6 × 10^1^ (1.0 × 10^1^–5.12 × 10^2^)^a^	8.1 × 10^3^ (1.47 × 10^1^–1.44 × 10^5^)^a^	5.0 × 10^3^ (1.0 × 10^2^–2.0 × 10^4^)^a^	1.9 × 10^5^ (2.2 × 10^3^–7.6 × 10^5^)^ab^
**56**	4.1 × 10^3^ (2.0 × 10^2^–1.37 × 10^4^)^a^	2.0 × 10^5^ (8.1 × 10^1^ -6.0 × 10^5^)^a^	2.0 × 10^5^ (8.7 × 10^3^–7.3 × 10^5^)^a^	4.5 × 10^5^ (1.6 × 10^4^–9.6 × 10^5^)^ab^
**Median**	4.47 × 10^2^	1.47 × 10^3^	1.44 × 10^4^	4.98 × 10^5^

#### BALF samples

All BALF samples from mock-inoculated animals were negative by qPCR during the whole study, while samples from inoculated animals were positive. Significant differences (*p* < 0.05) were only detected between the mean values at 14 dpi and 28 dpi. These results are shown in Table 2.

### Gross lung lesion scoring (GLLS) and microscopic lesion scoring in tracheal samples

Higher GLLS were seen at 14 dpi and 28 dpi than at 42 dpi and 56 dpi. Despite the numeric differences between means, no significant differences were detected using Tukey’s test (*p* < 0.05). Scar tissue and gray fish-flesh-like areas were noted at 42 dpi and 56 dpi. No gross lesions were observed in the lungs of the mock-inoculated group.

Results of cranial trachea lesion scores (CTLS), medium trachea microscopic lesion score (MTLS), lower trachea lesion score (LTLS), and GLLS are shown in Table 3. No significant differences were found in tracheal lesions scores between time points or anatomical regions when using the Tukey test or linear mixed-effects model test adjusted for Tukey (*p* < 0.05).

**Table 3:** Mean gross lung lesion score (GLLS) and microscopic lesion scoring (standard deviation) in samples from three different anatomical sites of the trachea at different days post-infection (DPI): Cranial trachea (CT), Medium trachea (MT), and Lower trachea (LT) of experimentally inoculated animals with *M. hyopneumoniae* strain 232. Lungs were scored using the Christensen et al. (1999) [17] methodology. No significant differences (*P* < 0.05) were found using Tukey test or linear mixed-effects model test adjusted for Tukey

*DPI*	*Control*				*Inoculated*			
	GLLS (%)	CT	MT	LT	GLLS (%)	CT	MT	LT
14	0.0 (0.0)	0.5(±0.5)	0 (0.0)	0 (0.0)	13.43 (2.21)	1.0 (0.82)	1.75 (1.7)	2.75 (1.5)
28	0.0 (0.0)	0 (±0.0)	0 (0.0)	0 (0.0)	13.22 (7.07)	2.25 (1.71)	1.75 (0.38)	2.75 (0.96)
42	0.0 (0.0)	0 (±0.0)	0 (0.0)	0 (0.0)	9.61 (7.0)	0.75 (0.5)	1.75 (0.5)	2.25 (1.5)
56	0.0 (0.0)	0.5 (±0.5)	0.5 (0.5)	0.5 (0.5)	8.75 (5.72)	1.5 (1.0)	3.0 (0.81)	3.0 (0.81)

#### *M. hyopneumoniae* detection in lung samples

All lung samples from inoculated animals were positive for *M. hyopneumoniae* using qPCR. In contrast, lung tissue samples from mock-inoculated animals did not show any positive results in the qPCR test.

### Correlations of *M. hyopneumoniae* load, GLLS, and tracheal sections lesion score

*M. hyopneumoniae* estimated load in BALF samples was significantly correlated with GLLS at 14 (0.93), 42 (0.95), and 56 dpi (0.89). Regarding tracheal samples, *M. hyopneumoniae* estimated load in CT was only correlated with GLLS at 28 dpi (0.86), while the load in MT correlated with GLLS at 28 (0.78) and 56 dpi (1.0) and with MTLS at 14 dpi (0.89). Finally, the load in LT was correlated with LTLS at 14 dpi (0.93). All coefficient values obtained for significant correlations and the significance values at all four time-points are shown in Table 4.

**Table 4:** Significant correlations coefficients (r) found between *M. hyopneumoniae* (MHP) quantification in trachea samples (Cranial trachea - CT; Medium trachea- MT and Lower trachea- LT) and bronchoalveolar lavage fluid (BALF) on the one hand and gross lung lesion score (GLLS) and microscopic lesion score in cranial (CTLS), medium (MTLS) and lower trachea (LTLS) on the other hand at different days post-infection (dpi)

**MHP quantification**	**14 dpi**				**28 dpi**				**42 dpi**				**56 dpi**			
	**GLLS**	**CTLS**	**MTLS**	**LTLS**	**GLLS**	**CTLS**	**MTLS**	**LTLS**	**GLLS**	**CTLS**	**MTLS**	**LTLS**	**GLLS**	**CTLS**	**MTLS**	**LTLS**
**CT**	–	–	–	–	0.86*	–	–	–	–	–	–	–	–	–	–	–
**MT**	–	–	0.89*	–	0.78*	–	–	–	–	–	–	–	1.0***	–	–	–
**LT**	–	–	–	0.93**	–	–	–	0.75*	–	–	–	–	–	–	–	–
**BALF**	0.93**	–	–	–	–	–	–	–	0.95**	–	–	–	0.89*	–	–	–

* *p* < 0.05 ** *p* < 0.01 *** *p* < 0.001

## Discussion

Understanding *M. hyopneumoniae* dynamics is essential for accurate diagnosis of infection, as well as for optimizing treatment and control measures in affected herds. Therefore, this study focused on reporting the infection dynamics upon experimental *M. hyopneumoniae* infection in specific sites (nasal swabs, CT, MT, LT, and bronchial tree) of the respiratory tract during a period of 56 dpi. To this end, *M. hyopneumoniae* DNA was quantified by qPCR in BALF and three anatomical sections of the trachea. In addition, gross Mycoplasma-like lung lesions and microscopic lesions of the trachea were scored.

Throughout the experimental period (0 to 56 dpi), the highest *M. hyopneumoniae* loads in tracheal sections (assessed by qPCR testing) were detected at 28 dpi (4.6 × 10^3^ copies∕μL) for CT, and at 56 dpi for MT (2.0 × 10^5^ copies∕μL) and LT (1.9 × 10^5^ copies∕μL) samples. Even though no statistical significance was found between *M. hyopneumoniae* quantification values in the tracheal sections, a numeric increase in *M. hyopneumoniae* load was observed in MT samples over time. Likewise, the load remained consistently high in LT samples throughout the whole studied period.

This pathogen is known for having tropism for lower parts of the swine’s respiratory tract, being up to 100 times more common in such sites [6]. Previous studies have also shown that *M. hyopneumoniae* is more accurately detected in samples collected from the lower respiratory tract; consequently, lower Cq values were obtained from deep tracheal swabs than from nasal and tonsil swab samples in a previous report [15]. A more recent study showed a significantly higher sensitivity for deep tracheal catheter (sensitivity range: 0.71–0.94) samples when compared to laryngeal swabs (0.27–0.57), mainly during late stages of infection (113 dpi) [12].

The abovementioned findings are in accordance with this study’s results, in which higher *M. hyopneumoniae* loads were reported in the medium and lower tracheal sections. This fact is mostly related to the better diagnostic sensitivity found in samples obtained from such anatomical regions, like the deep tracheal catheters [12, 13].

The first step of *M. hyopneumoniae* infection is adhesion to ciliated cells of trachea, bronchi, and bronchioles mucosal epithelial lining, leading to inflammatory cells’ infiltration and their accumulation around airways and blood vessels [18]. The lesions described above were also noted in tracheal samples from inoculated animals. Even though no significant differences were found, the scores seemed to increase over time, since the animals at 56 dpi presented the highest scores.

An in vitro inoculation of *M. hyopneumoniae* in cultured porcine tracheal cells showed progressive colonization and damage of the mucosal epithelial lining, noted from the 5th dpi onwards and with increasing severity over time [19], corroborating our microscopic lesion score results. In addition, a significant correlation between *M. hyopneumoniae* burden inoculated in swine tracheal cell culture with cilia loss in the same tissue [20], pathological alterations, and apoptosis [21], was reported, showing that the severity of the lesion may be affected by the pathogen infection load on-site.

In this research, LT samples showed numerically higher microscopic lesion scores when compared with MT and, especially, with CT. Interestingly, significant correlations were found at 14 dpi between *M. hyopneumoniae* load in MT (0.89) and at 14 and 28 dpi in LT (0.93 and 0.75, respectively) samples. Besides, as reported previously, the high load of *M. hyopneumoniae* found in these samples seems to play a role in the severity of microscopic lesion development in the first weeks of infection [11, 19].

Regarding the burden in BALF, the highest *M. hyopneumoniae* quantification value was reported at 28 dpi (1.2 × 10^6^ copies∕μL). Since it did not significantly decrease under the present conditions, the pathogen possibly could have persisted for a longer period and therefore not cleared from the body after 56 dpi. In addition, a previous study showed that all experimentally infected animals were positive up to the 94 dpi, and approximately 60% had positive bronchial swabs results at 214 dpi [22].

Interestingly, the *M. hyopneumoniae* load in BALF samples was significantly correlated with GLLS at 14, 42, and 56 dpi, highlighting the importance of its burden in lesion severity. Likewise, Vranckx et al. [2] reported a similar correlation between the pathogen load in lung lesion samples and GLLS.

In this study, the mean *M. hyopneumoniae* load in CT and GLLS reached the highest values at 28 dpi and were significantly correlated (0.86). Another study showed that *M. hyopneumoniae* load in nasal swab samples was significantly correlated to the load present in lung lesion homogenate of the same animal, showing that *M. hyopneumoniae* cells present in cranial regions of the respiratory tract are probably originated from lung lesions [6]. Therefore, it is possible to hypothesize that sampling the upper respiratory tract (i.e. nasal and laryngeal swabs) could result in the accurate detection of *M. hyopneumoniae*, considering the chronology of the disease. Nevertheless, further studies are necessary to achieve more accurate results regarding the association between diagnostic samples sensitivity and GLLS at the early stage of infection.

Additionally, on the 56 dpi, GLLS showed its lowest percentage (8.75%) while the LT pathogen load was still high. This result agrees with previous findings, which confirmed that sampling lower respiratory tract sites would be more accurate for *M. hyopneumoniae* detection in later or chronic stages of the disease [12, 13, 23] when clinical signs are mild, and GLLS is regressing.

*M. hyopneumoniae* was first detected in nasal swabs at seven dpi and presented the highest number of positive animals at 21–28 dpi (92 and 81%, respectively), allowing us to hypothesize that this was the time point when most animals were shedding the agent. Moreover, the detection of the pathogen in swab samples as early as five dpi and 28–30 dpi (peak) was already reported in other studies, as well as the intermittent detection pattern [6, 24, 25].

Unfortunately, the Monte Carlo effect [26] is an inherent limitation of the qPCR technique commonly seen in samples with a low target DNA copy concentration. In such samples, quantification cannot be accurately performed since the Cq values obtained between duplicates vary significantly. Thus, it is likely that this effect prevented us from properly quantify *M. hyopneumoniae* shedding in nasal swab samples.

The present results originated from an experimental infection using intratracheal inoculation of one particular strain of *M. hyopneumoniae* (232). The experimental infection model used in this study was successful since all inoculated pigs were confirmed to be positive through qPCR and developed *M. hyopneumoniae*-like lesions in the lungs, as well as associated clinical signs.

It is worth mentioning that the experimental infection conditions were different from those on the field, mainly because of the infecting dose, the pathogen inoculation site, and housing conditions (such as dust, humidity, and air circulation within the barn). Consequently, the presented results should be extrapolated to field animals with caution, even though some basic principles such as the dynamics of the infection and the *M. hyopneumoniae* burden in tracheal sites may be valid under practical situations. It is also fair to acknowledge that the limited number of animals used in each group could be associated with the wide range of quantification results reported within groups.

Finally, even though qPCR is a sensitive technique for detecting pathogens in biological samples, it is essential to mention that it detects not only live *M. hyopneumoniae* cells but also dead ones. Consequently, the quantification results could be slightly overestimated.

## Conclusion

The amount of *M. hyopneumoniae* genome fragments in the lower tracheal samples and BALF were high and remained relatively stable throughout 56 dpi. The CT samples’ load was numerically high at 28 dpi and associated with GLLS. Microscopic tracheal lesion scores seemed to increase with time and were more prominent in LT. To the best of our knowledge, this is the first study to experimentally assess the *M. hyopneumoniae* burden in different tracheal anatomical sections throughout 56 dpi, demonstrating the association of lung lesion and the pathogen dynamics in the respiratory tract.

## Methods

### Study design and experimental infection

A total of 24 male Large White pigs of 28-days of age were selected for this study. The animals originated from an *M. hyopneumoniae*, *Actinobacillus pleuropneumoniae,* and Porcine Reproductive and Respiratory Syndrome virus (PRRSv) free farm (since Brazil is a PRSS free country), being vaccinated only against swine influenza virus and porcine circovirus 2 (PCV2) with commercial vaccines. Moreover, the pigs remained without any noticeable clinical signs of infectious diseases until the inoculation. The animals were allocated in non-inoculated (*n* = 8) and inoculated (*n* = 16) groups. Each group was housed in isolated facilities (four animals per pen) with free access to water and fed twice a day, with an acclimatization period of 7 days before the inoculation. Laryngeal swabs and blood samples were collected on the arrival day (dpi − 7) and on the inoculation day (dpi0) to confirm the *M. hyopneumoniae* -free status by qPCR and ELISA (*Mhyo* atb test, IDEXX, USA), respectively.

At dpi 0, control animals (*n* = 8) were mock-inoculated with 10 mL of sterilized Friis medium. The inoculated animals (*n* = 16) were given 10 mL of macerated lung inoculum (from experimentally infected cesarean-derived colostrum-deprived pigs; obtained from Iowa State University) containing 10^6^ CCU∕mL of *M. hyopneumoniae* strain 232 (moderate virulence) diluted in sterilized Friis medium. Inoculation was performed with a laryngoscope’s aid to introduce a sterile catheter in the trachea until the trachea bifurcation (approximately 20 cm deep) [18]. After the inoculation, the catheter was rinsed with 10 mL of sterilized physiological solution to ensure that the inoculum was completely administered. Then, the animals were clinically examined daily to detect possible clinical signs related to *M. hyopneumoniae* infection.

Every 14 days, at 14 dpi, 28 dpi, 42, and 56 dpi, two control animals and four infected animals were randomly selected and euthanized according to the guidelines of the Brazilian Federal Veterinary Medicine council: 1 mg∕Kg bodyweight of Xylazine (Anasedan, Ceva) by intramuscular injection, followed by intracardiac injection of propofol (16 mg∕Kg) (Propovan, Cristália - Brazil). After the euthanasia, a necroscopic evaluation was performed, along with the score of gross lung lesions and the collection of biological samples for further analysis.

### Collection of nasal swabs, bronchoalveolar lavage fluid (BALF), lung, and tracheal samples

Nasal swabs were collected weekly using sterilized cotton swabs (Absorve, Brazil), which were inserted in both pig’s nostrils, rotated three times clockwise, placed into DNAse and RNAse free microtubes (Axygen, USA) containing 500 μL of sterilized Phosphate Buffered Saline (PBS) pH 7.4 (Sigma- Aldrich, USA), and kept at − 80 °C until processing.

During necropsy, the respiratory tract from the larynx to the lungs was removed from the carcass and placed on a separate table. BALF samples were collected by injecting 15 mL of sterilized PBS (pH 7.4) (Sigma-Aldrich, USA) in the bronchial bifurcation with the aid of a sterilized glass pipette and an automatic pipettor. The lungs were gently massaged, and then the PBS was pulled back, placed into 1.5 mL DNAse and RNAse free cryovials (Corning, USA), and immediately stored at − 80 °C until use.

Samples of gross cranio-ventral consolidation lesions, considered characteristic of *M. hyopneumoniae* infection, were collected to confirm its involvement in the lung lesions seen at the necropsy. Fragments of approximately 1 cm^3^ were collected in the transition area of affected and healthy tissue using sterilized scalpel and forceps, instantly placed in DNAse and RNAse free cryovials (Corning, USA), and stored at − 80 °C until use. Tissue samples of mock-inoculated animals were collected from all lung lobes since no gross lesions were seen during necropsy. For the inoculated animals with lesions in more than one lung region, samples were collected from all lesions and pooled into the same container.

Besides, tracheal samples from three different anatomic sites, namely, cranial trachea (CT) (from the larynx to the 12th tracheal ring), medium trachea (MT) (from 13th ring to the 24th tracheal ring), and lower trachea (LT) (from 25th tracheal ring until the bronchus bifurcation), were collected from each animal. Each tissue sample was divided into two; one was stored in individual DNAse and RNAse free microtubes (Axygen, USA) and immediately stored at − 80 °C while the other was stored in 10% buffered formalin for 24 h and later transferred to 70% alcohol.

### DNA extraction from biological samples and assessment of PCR inhibitor presence

BALF samples were initially centrifuged (Centrifuge 5804 R, Eppendorf, Germany) at 13,000 *g,* at 4 °C for 20 min. The sample’s supernatant was discarded, and the pellet resuspended in 250 μL of sterilized DNase and RNase-free PBS (pH: 7.4; Sigma-Aldrich, USA). To extract the DNA from tissue samples, 0.5 g of lung tissue and 0.2 g of trachea tissue were used. Regarding DNA extraction from liquid samples, 250 μL of resuspended BALF pellet and 500 μL of PBS from nasal and laryngeal swabs were used in an in-house protocol previously published [27]. The concentration and purity of the extracted samples were assessed through spectrophotometry (Nanodrop®, Thermo Fisher- USA). Overall, tissue samples presented a similar DNA concentration (900 ng∕μL), but the ones with different value were either diluted or re-extracted to obtain an equal DNA concentration in each sample. The DNA samples were stored in 200 μL microtubes DNAse and RNAse-free (Axygen, USA) and kept at − 20 °C until use. The presence of PCR inhibitors in the extracted DNA was assessed by performing a conventional PCR protocol for the endogenous gene of Glyceraldehyde-3-Phosphate Dehydrogenase (*gapdh)* gene, using a previously published protocol [28]. To get tested with qPCR, the DNA samples had to be positive in the conventional PCR targeting the *gapdh* gene.

### Detection and absolute quantification of *M. hyopneumoniae* by qPCR in biological samples

*M. hyopneumoniae* DNA detection was performed with the aid of qPCR in tracheal and lung samples, nasal swabs, and BALF. All samples were tested in duplicates, using *M. hyopneumoniae* specific primer pair and hydrolysis probe from a previously published protocol [29].

The reaction was composed of 1X Master mix Go taq® (Promega, Madison, USA), 0.5 μM of each primer (Invitrogen, USA), 0.3 μM of the hydrolysis probe (IDT, Iowa City- USA), ultrapure water q.s.p and 1 μL of DNA template, adding up to a final reaction volume of 10 μL. The real-time thermocycler used was a CFX-96 (Biorad - USA) model. The cycling conditions were: one cycle of initial denaturation at 95 °C for 3 min, followed by 39 cycles of 95 °C for 15 s, and annealing∕extension at 55.7 °C for 1 min. Quantification results were only used if the Cq difference was lower than 0.5 cycle [16]. In case of Cq difference higher than 0.5 cycle, the samples were tested again in triplicates.

Absolute quantification and detection limits were performed using standard curves. The curve consisted of duplicate 10-fold dilutions, starting at 10^7^ copies∕μL until 10^1^ copies∕μL, of a synthetic DNA positive control (GBlock®, IDT, Iowa City, IA, USA) of the 150 bp amplified fragment. In addition, quantification data was only used if qPCR efficiency was between 90 and 105% [30].

### Gross lung lesion scoring

Lungs of both inoculated and control animals were scored (before tissue sample collection) for characteristic lesions of *M. hyopneumoniae* infection, like cranio-ventral consolidation lesions in the apical, intermediate, accessory lobes and the cranial part of diaphragmatic lobes. Briefly, each of the lobes was visually assessed, and the percentage of the affected area was estimated and further multiplied by the correction factor, based on each lobe’s proportional weight in the total lung weight, as described previously [17]. Gross lesions that were not characteristic of *M. hyopneumoniae* infection were not scored in this study.

### Microscopic lesion scoring in the tracheal samples

Tracheal samples were submitted to standard histopathology processing. The slides were then stained with Hematoxylin-Eosin and evaluated using a light microscope. The scoring process of the microscopic lesion was based on the description of the *M. hyopneumoniae* characteristic microscopic lesions in tracheas [31]. Lesions were scored from 0 to 4 according to the criteria shown in Table 5. A blinded pathologist performed the lesion scoring by checking ten random microscopy fields.

**Table 5:** Microscopic lesion grading criteria adopted for scoring tracheal samples of *Mycoplasma hyopneumoniae* (strain 232) experimentally infected pigs

**Score**	**Lesion description**
**0**	No lesion
**1**	Epithelium hyperplasia, ciliated cells present in standard quantity with dense cilia
**2**	Epithelium hyperplasia, reduced cilia density and mild presence of hemorrhage
**3**	Epithelium hyperplasia with largely reduced cilia density, glandular hyperplasia and increased number of goblet cells
**4**	Epithelium hyperplasia, glandular hyperplasia, increased number of goblet cells and presence of inflammatory cells infiltration

### Data analysis

All numeric data was analyzed using the R software (R Core Team, 2018). Data normality was assessed through the Shapiro-Wilk (SW) test, and homoscedasticity was tested using the Bartlett test, both with 95% confidence level (*p* < 0.05). Significant differences in parametric data were tested through analysis of variance (ANOVA), and multiple pairwise comparisons were made using the Tukey test and Duncan test (*p* < 0.05). Significant differences in non-parametric data were assessed through Wilcoxon and Kruskal-Wallis tests, and multiple comparisons between data sets were made by using Dunn’s test (*p* < 0.05). For non-parametric comparison of repeated measurements, the Friedmann test was used (*p* < 0.05), while for parametric datasets a linear mixed-effects models adjusted for Tukey (*p* < 0.05) were used. Correlations were tested using Pearson’s correlation (parametric models) test or Spearman’s correlation rank test (non-parametric models).

## Data Availability

The datasets used and/or analyzed during the current study are available from the corresponding author on reasonable request.
